# An infertile female delivered a baby after removal of primary renal carcinoid tumor

**DOI:** 10.1515/med-2020-0408

**Published:** 2021-01-18

**Authors:** Syu Jhang, Allen W. Chiu

**Affiliations:** Department of Urology, MacKay Memorial Hospital, No. 92, Sec. 2, Zhongshan N. Rd., 10449, Taipei, Taiwan; School of Medicine, Mackay Medical College, No. 92, Sec. 2, Zhongshan N. Rd., 10449, Sanzhi, Taiwan; Division of Urology, Department of Surgery, Taipei City Hospital, No. 92, Sec. 2, Zhongshan N. Rd., 10449, Taipei, Taiwan; School of Medicine, National Yang-Ming University, No. 92, Sec. 2, Zhongshan N. Rd., 10449, Taipei, Taiwan

**Keywords:** carcinoid tumor, infertility, neuroendocrine tumor, primary carcinoid tumor of the kidney, renal carcinoid tumor

## Abstract

Primary renal carcinoid tumors are exceedingly rare. We report a 37-year-old woman with primary infertility, who was found to have a primary renal carcinoid tumor. She became pregnant and gave birth to a baby after removal of the tumor. This is the first case in the English literature of primary renal carcinoid tumor related with primary infertility.

## 1 Introduction

Carcinoid tumors are neoplasms arising from neuroendocrine cells. Approximately 100 cases of primary renal carcinoid tumors have been documented in the English literature [1,2]. We report the first case of primary renal carcinoid tumor associated with primary infertility.

## 2 Case report

A woman aged 37 years was referred to our department after being diagnosed incidentally with a 3.3 cm left renal tumor on abdominal sonography. She had been married for 4 years. She failed to conceive with regular unprotected sexual intercourse for 3 years. She visited the clinic of obstetrics and gynecology for infertility without effect 1 year ago.

**Figure 1:**
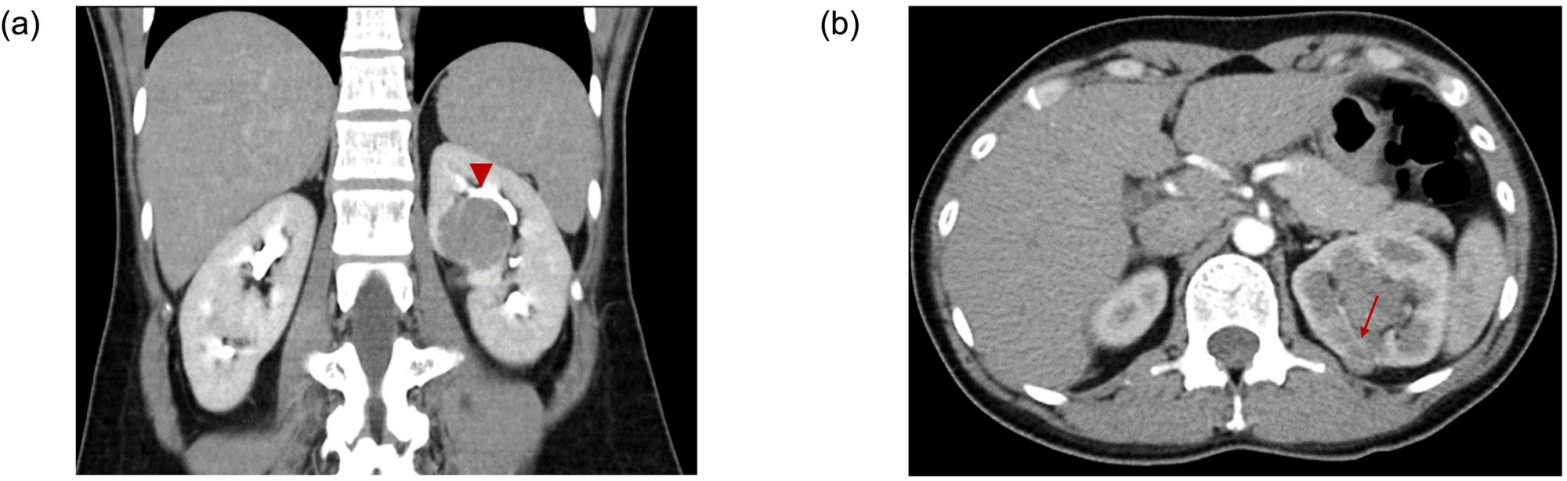
Abdominal CT revealed (a) the main endophytic tumor (arrow head) in the dorsal interpolar region of left kidney and (b) another exophytic tumor (arrow).

She had no other symptoms except palpitation, intermittent facial flushing and sweating for 8 years. These symptoms became more frequent in recent 6 months. Laboratory tests including T3, T4, TSH, prolactin, FSH, LH, estradiol and DHEA-S were within normal range. The semen analysis of her husband revealed no abnormality. Hysterosalpingography showed bilateral patent fallopian tubes. Transvaginal ultrasound revealed mild polycystic ovary disease. Abdominal computed tomography revealed a 3.8 × 3.8 × 3.2 cm endophytic renal mass with mild contrast enhancement in the dorsal interpolar region of the left kidney. There was another 1.3 × 1.0 × 0.5 cm exophytic renal mass (Figure 1).

**Figure 2:**
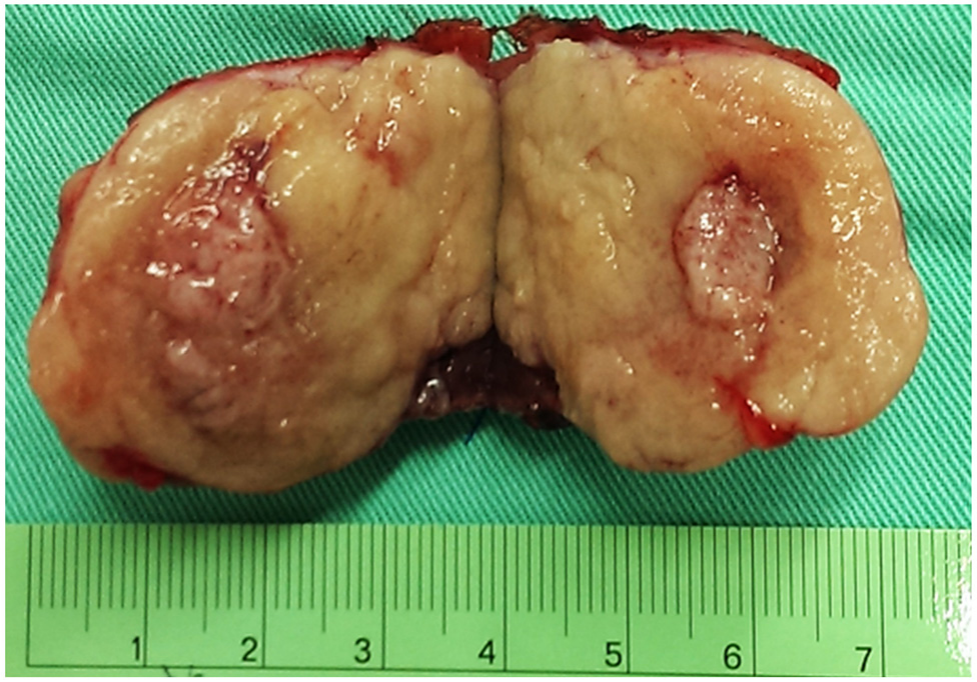
The specimen of the main renal tumor.

**Figure 3:**
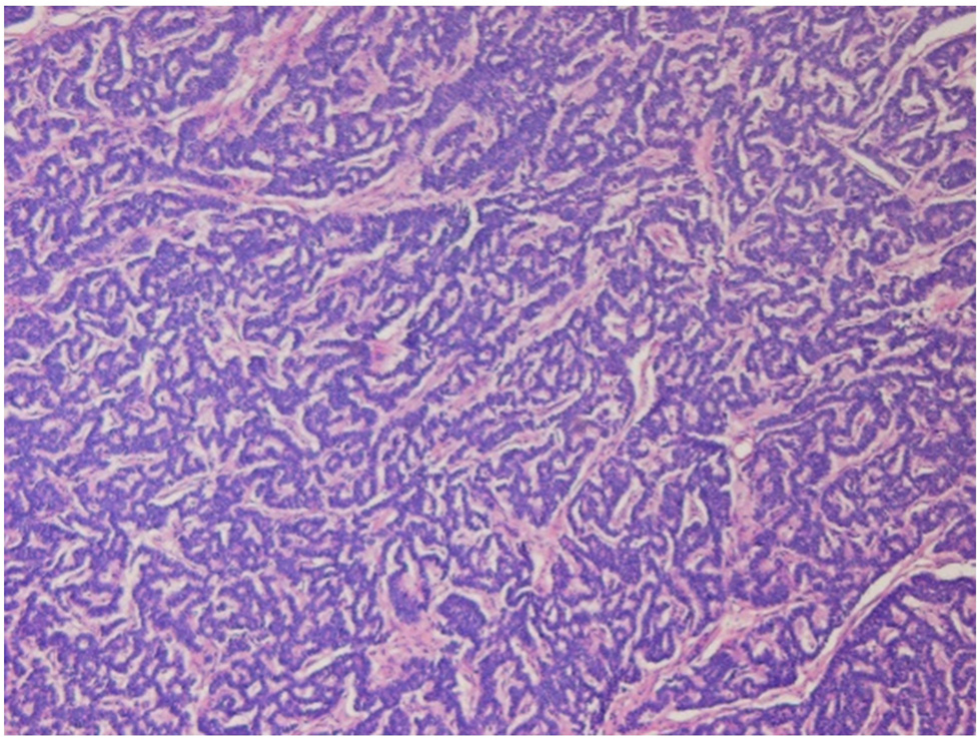
Microscopic findings of H&E staining showed that the tumor grew in a gyriform pattern with hyperchromatic nuclei. NET, neuroendocrine tumor.

Given the uncertain nature of the tumors, we first excised the exophytic renal tumor and checked the tumor nature with intraoperative frozen section for tissue proof. As there was no evidence of malignancy of the frozen section, we then performed enucleation of the main endophytic tumor instead of radical nephrectomy (Figure 2). Microscopic finding revealed the tumor having uniform and hyperchromatic nuclei, growing in a gyriform and ribbon-like pattern without a well-formed capsule (Figure 3). Immunohistologically, the tumor cells expressed neuroendocrine markers including CK, vimentin, neuron-specific enolase, synaptophysin and chromogranin. The final diagnosis is a well-differentiated, grade 1, neuroendocrine tumor (NET), also known as a carcinoid tumor.

After the surgery, the symptoms including palpitation, facial flushing and sweating disappeared. Six months after the surgery, the patient received intrauterine insemination. She became pregnant and delivered a healthy baby.

## 3 Discussion

Carcinoid tumors, one of NETs, are neoplasms usually originating in the gastrointestinal tract [3,4]. Primary carcinoid tumors of the kidneys are extremely rare. We conducted a PubMed search to find published research in August 2018, using the keywords: “primary carcinoid tumor” and “kidney.” There has been about 100 cases reported in the English literature [1,2].

A total of 25–30% of primary renal carcinoid tumors are found incidentally [2]. In symptomatic patients, the most commonly reported symptom was abdominal or flank pain [4,5]. Only 12.7% of patients with primary renal carcinoid tumors present with carcinoid syndrome, characterized by flushing, generalized edema, nausea, vomiting and diarrhea [5]. Our patient belonged to the symptomatic group. The symptoms subsided after the surgery. Interestingly, she became pregnant 6 months after the surgery and gave birth to a healthy baby, which may be related with the removal of primary renal carcinoid tumor. Swelstad et al. reported a case of incidentally identified NET of the appendix, which was associated with the primary infertility [6]. However, they did not report whether the female patient became pregnant.

To our knowledge, this is the first such case. Despite reports in women with primary carcinoid tumor in the kidney [7] and in other locations [8,9] still achieving spontaneous pregnancy, we cannot assume that carcinoid tumors have no negative impact on conception. Although the mechanisms of how NETs influence the conception are still unknown, an endocrinologic rationale for infertility in NETs has been proposed. Serotonin, considered as the cause of carcinoid syndrome, has shown to reduce the uterine blood flow and affect fetal nutrition to cause fetal death in animal studies [7]. Selective serotonin reuptake inhibitors, which elevate the level of serotonin, typically prescribed as antidepressants, have been shown to increase the risk of spontaneous abortion during pregnancy [10].

In conclusion, we present the first case of an infertile female delivering a baby after removal of primary renal carcinoid tumor. Further investigation is needed to know the mechanisms of how NETs influence the conception.
